# Is trust built on spirit? Examining spiritual leadership as a multidimensional predictor of organizational trust; pragmatic evidence from Malaysian public universities

**DOI:** 10.3389/fpsyg.2025.1617461

**Published:** 2025-10-24

**Authors:** Zulkiflee Daud, Hosam Azat Elsaman, Mazli Mutazam, Mohd Rashdan Sallehuddin, Nasiruddin Haron, Mohd Abidzar Zainol Abidin, Rusli Ahmad

**Affiliations:** ^1^College of Business, Northern University of Malaysia, Sintok, Malaysia; ^2^Sohar University, Sohar, Oman

**Keywords:** spiritual leadership, organizational trust, public university, Malaysia, conceptual model validation

## Abstract

A lack of organizational trust has been consistently linked to deteriorating performance outcomes in academic institutions, particularly in administration staff in the Malaysian higher education sector, when trust is deficient that can lead to reduce the staff morale and negatively affect their motivation, this directly impact the whole organizational performance. This study examined the effect of spiritual leadership on organizational trust amongst administrative and support staff in a public university in Malaysia. The authors developed a conceptual model to interpret the relation between spiritual leadership and organizational trust. Stratified random sampling technique was used in data collection, with a sample size of 306 and data analyzed using statistical package of social science SPSS, thus, the study executed factor analysis and multiple regression analysis in validating and statistically detecting the significant effect between the spiritual leadership which significantly predicted and interpreted the organizational trust. The study variable hope is significantly influenced by all three dimensions of organizational trust which are leader’s reliance, leader’s behavior and harmony. This study contributes to the literature on how spiritual leadership influence the organizational trust particularly in public universities in Malaysia.

## 1 Introduction

The concept of “trust” in social science generally retains its everyday meaning, primarily referring to the level of a person’s willingness to trust the good deeds of others and have faith in their words and actions (Kark and Shamir, 2023). Trust within an organization can be manifested in various ways, it can develop among colleagues, as well as in the feeling of the employees toward the company and its leadership. Additionally, trust can span across different departments and even between distinct organizations.

Daud et al. (2019) observe that most definitions of trust are grounded in an individual’s expectations about another person’s future behavior, shaped by that person’s current and past statements both explicit and implicit about how they will act, and the implicit claims often arise from an individual’s cooperative behavior, which indicates a crucial role in influencing how we perceive others.

Organizational trust is the positive belief that individuals have regarding the intentions and actions of various members within the organization, shaped by roles, relationships, experiences, and interdependencies. It is a general assessment of an organization’s dependability, as well as the level of support and trust employees feel toward their employer. Current research suggests that employees’ perceptions of the organization’s core trust are crucial for sustaining trust-building practices (Daud et al., 2018). Therefore, organizational trust reflects employees’ confidence in their direct supervisors, as well as the trust among employees, management, work units, and team members. Organizational trust also pertains to interpersonal trust among workgroups or teams. This indicates that a network of trust influences participation in corporate decision-making, crisis management, conflict resolution, and the mitigation of disputes (Moghavvemi et al., 2018).

A lack of organizational trust has been consistently linked to deteriorating performance outcomes in academic institutions, particularly in administration staff in the Malaysian higher education sector, since trust acts as a foundational element for effective communication, collaboration and the psychological safety of university staff, moreover, when trust is deficient that can lead to reduce the staff morale and negatively affect their motivation, this directly impacts the overall organizational performance (Saraih et al., 2018). In the Malaysian public university context studies show that diminished trust in leadership correlates with reduced work engagement and job satisfaction among administrative and support staff, this erosion of trust can inhibit the innovation and limit constructive dialogue, also ultimately impair institutional effectiveness (Abbasi and Wan Ismail, 2023). According to Hassan and Ahmed (2011), the absence of leader credibility and transparency fosters a culture of fear and compliance rather than empowerment, undermining the collective commitment required for academic excellence. Moreover, less trust environments are characterized by fragmented teamwork and resistance to change, both of which are detrimental to institutional transformation initiatives. This is especially problematic in Malaysian academia where hierarchical structures are dominant and trust-building mechanisms are often underdeveloped, therefore, enhancing organizational trust is not merely a relational concern more than it is a strategic imperative for improving performance, fostering collaboration and achieving sustainable growth within Malaysian universities (Ayduğ, 2025).

## 2 Literature review

Organizational trust significantly influences the retention of academic staff in Malaysian higher education institutions. A study by Basit and Duygulu (2018) highlights that trust in the organization is a critical factor affecting the intention of self-initiated academic expatriates to remain in Malaysian universities, the research indicates that when expatriate academics perceive a lack of trust from their institutions, it adversely impacts their commitment and increases turnover intentions. This finding spotted the importance of adopting a trustworthy environment to retain valuable academic talent.

Furthermore, trust plays a crucial role in promoting knowledge sharing behaviors among academics. Mutahar et al. (2022) found that trust among academic staff is essential for effective knowledge sharing, which is vital for academic excellence. The study emphasizes that in the absence of trust, academics are less likely to engage in knowledge-donating and collecting behaviors, thereby hindering collaborative efforts and innovation within the institution. These insights collectively suggest that cultivating organizational trust is not merely beneficial but essential for the sustainability and advancement of academic institutions in Malaysia.

Trust is essential for building strong relationships, deepening human connections, and providing a stable foundation for meaning and growth. In the workplace, cultivating trust involves fostering a collective belief in leaders who are dedicated, empathetic, and capable (Daud et al., 2023). Employees who trust their leaders are more likely to trust that decisions will focus on promoting the overall wellbeing. Moreover, employees highly value transparency from their leaders, especially during challenging times. This mutual trust fosters increased collaboration, loyalty, and overall confidence in the organization (Tsai and Wu, 2024; Daud et al., 2024b).

Building organizational trust is not particularly difficult. Leaders simply need to treat people fairly and clearly explain the rationale behind any changes. Effective communication is key in any organization; it should be authentic, consistent, and ideally two-way. A trustworthy leader motivates, educates, and empowers others through their communication (Elsaman et al., 2024a,b). Furthermore, messages should be engaging and customized to ensure that all recipients fully grasp their expectations.

While demonstrating competence is crucial for building trust, qualities like empathy and understanding can have an even greater impact. Leaders can further cultivate trust by embodying fairness, follow-through, and compassion. When business leaders commit to these values through equitable and impartial practices, they cultivate an environment where everyone feels valued, respected, and connected, ultimately promoting a stronger and more united workplace.

Research conducted by Vesudevan and Abdullah (2024) indicated that group cohesiveness and communication are key precursors of organizational trust. On the other hand, Tzafrir and Dolan (2004) designed a scale to measure organizational trust and identified three major antecedents: harmony, concern, and reliability. Reliability involves consistent and systematic practices and behaviors and is strengthened when promises and commitments are fulfilled. Concerning this matter, it transcends mere opportunism to encompass altruistic behavior, which illustrates another dimension of trust. Ultimately, a constructive amalgamation of emotions, interests, perspectives, objectives, and values inside the employment relationship framework fosters harmony. Mayer et al. (1995) proposed three critical characteristics of organizational trust in their study on an integrated model: capacity, benevolence, and integrity. Ability encompasses the collection of abilities, talents, and traits that enable an individual or entity to exercise influence within a specific domain. Benevolence refers to the extent to which a trustee is seen as genuinely concerned for the wellbeing of the trustor, free from self-interested motives. Integrity encompasses the employee’s perception of a set of standards deemed acceptable by the superior. Shockley-Zalabak et al. (2000) identified five significant antecedents of organizational trust in their study involving 10–14 focus groups of employees from the United States and Italy including openness and honesty, concern for employees, reliability, competence, and identification.

It is worth noting that many researchers who have studied the impact of trust in organizational performances specially in Malaysia adopted the qualitative approach, this highlight the need of conducting an empirical study about this point to clarify and validate the results of previous studies. In the same context many studies demonstrates that organizational trust is a critical determinant of employee wellbeing in academic and institutional settings. When trust is absent, employee outcomes such as job satisfaction, commitment and mental health are negatively affected.

Cheng and Wang (2025) argued that organizational trust significantly influences affective commitment and organizational behaviors, mediated by attitudinal pride. Similarly, Jaškevičiūtė and Šilingienė (2021) demonstrated that organizational trust acts as a vital mediator between human resource management practices and employee wellbeing and emphasizing its centrality in sustainable organizational systems. In addition, Yıldız and Şimşek (2019) reported a strong positive relationship between organizational trust and job satisfaction among Turkish physicians, underscoring its impact across institutional contexts. Moreover, Lee and Kim (2022) observed that trust enhances job satisfaction and lowers turnover intentions, positioning it as a protective factor against organizational disengagement. On the study of Chen and Huang (2016). These findings collectively indicate that trust is not only a moral imperative but a structural necessity in academic institutions. Failure to cultivate trust undermines institutional health, while its presence enhances performance, retention, and innovation across faculty and staff cohorts.

Based on these findings, the researchers realized the need of proposing the solutions to tackle this phenomenon by developing and validating a model to predict trust in organization through spiritual leadership.

Spiritual leadership is grounded in principles such as integrity, virtue, collaboration, knowledge, integration, and connectivity (Samul, 2024; Daud et al., 2021). Organizations that are deeply rooted in spirituality foster innovation and creative thinking among employees, as spirituality plays a vital role in driving creativity and innovation (Jahroni et al., 2024). In the process of creating a shared vision, spiritual leadership focuses on prioritizing collective goals over personal ambitions, aligning followers’ aspirations with the organization’s broader future, while also maintaining their trust and confidence by nurturing their hope and faith. This study aims to examine the impact of spiritual leadership on organizational trust (Sihombing et al., 2024).

Spiritual leadership is fundamentally an individual trait that influences organizational members to attain collective objectives. Effective leaders motivate and guide the organization and its members to actively pursue the set goals (Vedula and Agrawal, 2023). The leader’s influence in steering the organization toward its goals will build confidence among members, encouraging them to rely on and follow the guidance given. As defined, trust is the readiness to depend on an authority embedded in favorable anticipations regarding the actions and intentions of that authority, hence the robust trust of members in companies is influenced by the leader’s capacity to motivate them to execute their jobs efficiently (Chen et al., 2024).

Earlier research on leadership mainly concentrated on transformational and transactional leadership approaches, nevertheless, recently numerous instances of wrongdoing and mismanagement by management staff have emerged this necessitating an examination of the phenomena by utilizing and implementing the spiritual perspective in leadership within organizations (Constantin, 2024). According to Fry (2003), previously, leadership theories have traditionally concentrated on the physical, mental, or emotional aspects of human interaction within organizations, often overlooking the spiritual dimensions. On the other hand, it is grounded in a spirit-centered, value-driven approach, defined by the values, attitudes, and behaviors that are key to intrinsically motivating oneself and others. This approach fosters a sense of spiritual wellbeing through a deep sense of calling and belonging, In the same context a spiritual leader is an individual who genuinely encourages and inspires employees to cultivate vision, value, and a feeling of duty through spiritual direction, hence enhancing overall employee wellbeing (Fry, 2003; Samul, 2024).

Spiritual leadership represents a transformative approach to leadership studies. Rooted in “spirituality,” it is seen as a fundamental force that gives deep meaning to our human experience (Ghasemy et al., 2022). It acts as a universal energy that propels us to rise to new heights and build meaningful connections with others. The aim of spiritual leadership is to construct a shared vision and align values within employees or organization, acting as the force that unites individuals in purpose and harmony. Spiritual leadership is key in promoting proactive behavior in the workplace, highlighting the critical role of leadership in nurturing such behaviors (Hu and Zhang, 2023). As a result, managers are encouraged to adopt a spiritual leadership style that inspires their teams to become more engaged, committed, and productive, leading to greater proactive behavior in the workplace (Mutazam and Daud, 2021; Daud et al., 2019, 2024a).

Spiritual leadership is an inwardly cultivated characteristic. The leader’s character, values, and personality are shown. A leader who embodies spiritual leadership places greater emphasis on fostering harmonious and meaningful relationships with subordinates. According to Garg and Jalan (2024), leaders exhibiting spiritual leadership emphasize compassion, ethics, and human values in fostering meaningful relationships. Leaders should articulate their vision while delegating responsibilities to subordinates, thereby clarifying the expected outcomes to mitigate uncertainty and enhance subordinates’ sense of belonging. Therefore, examining spiritual leadership will enhance comprehension of the organization’s culture (Subhaktiyasa et al., 2023).

Spiritual leaders consistently employ emotional intelligence in their leadership. Emotional intelligence is the capacity to recognize, regulate, and effectively utilize one’s emotions to reduce stress, communicate clearly, empathize with others, solve problems, and resolve conflicts. Consequently, leaders exhibiting spiritual leadership will demonstrate honesty in their work, accountability in their decisions, fairness in their treatment of employees, and adherence to their commitments. According to the findings of Li and Wang (2023) leaders who engage in spiritual leadership will augment employee loyalty.

As maintained by Conway (1999), task performance refers to a defined set of behaviors focused on accomplishing work tasks, which directly impact how a supervisor assesses an employee’s overall contribution to the organization. Research shows a positive link between spiritual leadership and individual task performance and as mentioned by Guillen et al. (2015), spiritual leadership is considered an effective strategy for boosting employees’ intrinsic motivation by addressing the psychological needs of both leaders and followers, while also fulfilling essential spiritual needs, such as values and management practices that motivate individuals to engage in meaningful and purposeful work (Fry, 2003). Deci and Ryan (2000) indicated that intrinsic motivation is fueled by personal interest and fundamental psychological needs, which are expressed as independence, skill mastery, and connection in the workplace. Moreover, as maintained by Deci and Ryan (2000), intrinsic motivation is more likely to thrive in environments that are marked by warmth and compassion. The purpose of spiritual leadership is to intrinsically motivate individuals by embodying spiritual values and showing selfless compassion in the workplace, with the ultimate objective of boosting productivity (Fry, 2003; Fry et al., 2005; Fry and Cohen, 2009). According to Faruk et al. (2019a,b), many studies have demonstrated that intrinsic motivation is related with enhanced performance and learning.

Followers of leaders who practice spiritual leadership tend to perform better, driven by a shared and clear vision. Spiritual leadership inspires followers by presenting a compelling long-term vision and an alternate future. Locke et al. (1981) said clearly defined and challenging goals are more likely to enhance individual task performance. In fact, as mentioned by Locke et al. (1981), Joo et al. (2023), clear and challenging goals result in better performance compared to having no specific objectives. Through a vision grounded in spirituality, spiritual leadership instills faith and hope, guiding followers in the vision-creation process. A confident role model encourages followers to demonstrate perseverance and strive for excellence when tackling difficult tasks.

Spiritual leaders assist followers in nurturing their body, mind, heart, and soul. By crafting a compelling vision and mission, they can foster collaboration, trust, mutual support, and commitment. They gain the acceptance of followers by showing a deep understanding of the group’s dynamics and proving their expertise in areas such as credibility, guidance, trust, and inspiration. Several studies, including those by Terzi et al. (2020), Esfahani and Sedaghat (2015), have applied the spiritual leadership dimensions developed by Fry (2003). In his foundational paper “Toward a Theory of Spiritual Leadership,” Fry (2003) identifies three key attributes of spiritual leadership namely vision, altruistic love, and hope. These characteristics are seen as crucial for addressing followers’ need for spiritual survival, which in turn can influence organizational success. Fry (2003) argued that a leader’s vision provides clarity on the direction employees should follow for organizational sustainability, creating a meaningful existence that makes an impact. altruistic love, on the other hand, reflects a leader’s dedication to employee wellbeing, respect for their rights, and acknowledgment of their accomplishments. The third dimension, hope or faith, refers to how leaders communicate their vision, inspiring followers to have confidence in and be dedicated to the organization’s goals.

The global public sector confronts a common challenge: integrity, and currently, public sector reform is not particularly effective (Fourie and Poggenpoel, 2016). This assertion aligns with the perspective of Blum et al. (2012) that most countries possess sound underpinnings for effective governance but falter in implementation. In Malaysia, the integrity of public personnel is consistently a significant topic of concern. Rahmatika et al. (2022), noted that problems such as lack of trust, misuse of power, and misappropriation of public funds are consistently emphasized, adversely impacting the overall perception of the public sector. In his address, Ismail et al. (2019), stated that trust issues is the sole subject that has garnered extensive discussion. Consequently, all government organizations, including public universities, require competent officials to facilitate an effective governance structure. Faruk et al. (2019a,b) asserted that public universities require not just esteemed academicians but also proficient administrative officers capable of adeptly navigating the myriad hurdles encountered in their pursuit of becoming world-class institutions.

Hence, this study main objective: to examine the impact of spiritual leadership represents by dimensions namely vision, altruistic love, and hope/faith on organizational trust that represents by dimensions namely harmony, reliability and concern.

Based on a review of recent and existing literature, the research hypothesis is:

H1: The spiritual leadership has an influence on organizational trust.

**FIGURE 1:**
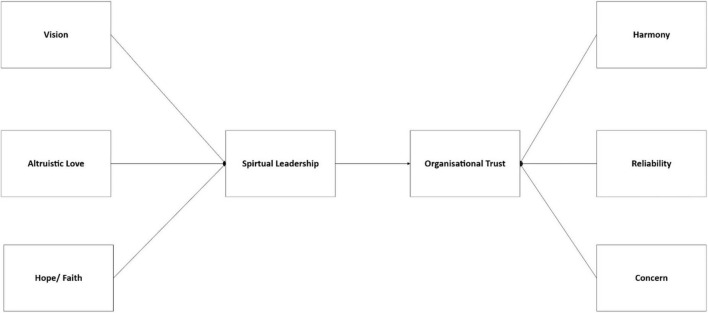
The research framework developed by authors.

As shown in Figure 1, the research framework is developed to include three dimensions of spiritual leadership and three dimensions of organizational trust.

## 3 Research methodology

The study employs a quantitative methodology by utilizing a Likert scale questionnaire-based approach to collect data from participants. A cross-sectional research design was employed for this study. The unit of analysis for this study is the individual, encompassing support staff a public university located at Northern Peninsular Malaysia.

### 3.1 Population and sample

This study involved 1,470 support staff members ranging from grade 11 to grade 36 across various departments and colleges. There are 30 departments, and seven colleges participated in this research. Form 1,470 total of population, according to Table of Proportionate Sampling established by Kriejcie and Morgan (1970), 306 samples were selected by using stratified proportionate sampling method. Respondents have thirty minutes to complete the questionnaire and submit it to the researcher upon finishing their answers. This approach is referred to as a self-administered questionnaire. A self-administered questionnaire is an effective method for data collection, as it allows researchers to review participant information promptly and at the respondents’ convenience, without hindering their work productivity. This study asserted that the questionnaire encompassed a substantial participant sample and provided clear information for conducting the current investigation. The researcher managed to finish the questionnaire distribution and collection process within 2 months. This study implemented parametric analysis with a probability sampling approach as a method wherein each member of the population possesses a known, non-zero likelihood of being selected. Every individual in the population has an identical likelihood of selection (Sekaran, 2003). A total of samples is obtained using a proportional stratified sampling method.

### 3.2 Instrumentation

The data for this research was mainly gathered through a questionnaire distributed to respondents in order to collect relevant information. The questionnaire, developed by the researcher based on previous studies including 16 items assessing organizational trust, taken from Tzafrir and Dolan (2004) and 17 items taken from Fry et al. (2005) to measure spiritual leadership.

Sixteen items were altered from Tzafrir and Dolan (2004) to evaluate the construct of organizational trust. This measurement encompasses three dimensions: harmony, reliability, and concern. Examples of items in this scale include, “The needs and desires of managers/employees are highly significant to employees/managers,” “I can rely on my colleagues and management for assistance during job-related challenges,” and “they would not intentionally engage in actions detrimental to the organization.”

As for measuring spiritual leadership, seventeen items were altered by Fry et al. (2005) to evaluate the construct of spiritual leadership. This test has three dimensions: vision (questions 1–5), hope or faith (questions 6–10), and altruistic love (questions 11–17). Examples of items on this scale include: “I comprehend and am dedicated to my organization’s vision,” “I possess confidence in my organization and am prepared to do whatever is necessary to ensure it achieves its mission,” and “My organization genuinely values its personnel.”

### 3.3 Data analysis technique

The data received from respondents via the questionnaire analyzed with Statistical Package for the Social Sciences (SPSS) software. This study aims to investigate the impact of spiritual leadership on organizational trust; hence the authors conducted a reliability test to assess the accuracy of participants responses to the questionnaire and to evaluate the interrelatedness of the items included, Cronbach’s alpha computes the average intercorrelations among the items utilized in this investigation to determine the reliability of the assessment. Elsaman et al. (2022) asserted that Cronbach’s alpha cutoff point of 0.7 is an acceptable threshold. Additionally, descriptive analysis was performed to provide information on the means, standard deviations, and frequencies of the main variables. Moreover, the correlation studies provide insights into the link between dependent and independent variables. Given that the correlation analysis provides inadequate insights into the relationship, multiple regression performed as the last analysis to determine the impact of spiritual leadership on organizational trust.

On the meantime, hypothesis test conducted by utilizing regression analysis, with a *p*-value of < 0.05 being significant (Sekaran and Bougie, 2010; Woo and Kim, 2020). Multiple regression is a proper test conducted to detect the relationship between independent and dependent variables. Hair et al. (2010) describes how a dependent variable can be (Hair et al., 2012) predicted by two or more independent variables, which shedding light on the nature of their relationship. The results from multiple regression revealed both the direction and significance of the connection between the variables. Additionally, regression analysis highlighted the influence of the independent variables on the dependent variable.

## 4 Results and findings

### 4.1 Reliability analysis result

Hair et al. (2006) stated that Cronbach alpha 0.70 value as the minimum cutoff standard, this presents as the benchmark for this test. **T**he Cronbach’s Alpha results for each variable in the current study are as follows: Organizational Trust was assessed using all 16 questions, yielding a Cronbach’s α value of 0.920. The 17 components of Spiritual Leadership yielded a Cronbach’s α value of 0.756 as shown in Table 1.

**TABLE 1:** Reliability test.

Variable	No. of items	Cronbach alpha value
Organizational trust	16	0.920
Spiritual leadership	17	0.756

### 4.2 Factor analysis

#### 4.2.1 Factor analysis for organizational trust

**FIGURE 2:**
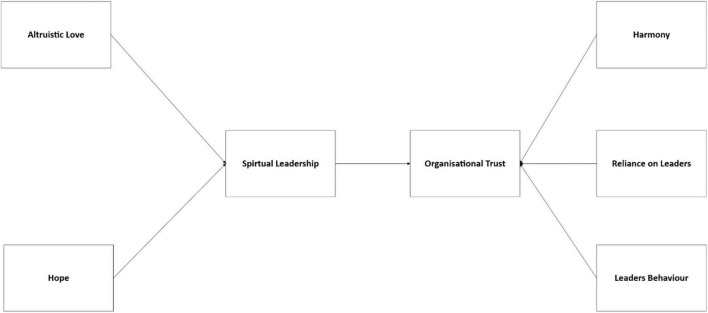
The research framework after factor analysis.

As shown in Figure 2, the results indicate that three factors were derived from the Rotated Component Matrix. The initial element comprises six items, including “My leader’s needs and desires are highly significant to employees,” “I can rely on my leader for assistance when facing job-related challenges,” and “My leader would not intentionally harm the organization.” The second element has five items including “I believe that individuals in the organization achieve success by undermining others,” “My leader articulates his/her genuine sentiments regarding significant matters,” and “My leader possesses extensive knowledge about the tasks that must be accomplished.” The third element has four statements, including “My leader’s actions and behaviors are inconsistent” and “My leader would make personal sacrifices for our group.” All items are subjected to a reliability test across all factors. The outcome indicates that no items were eliminated from this section, as all satisfied the reliability criteria and were adequate.

Table 2 above presents the Cronbach’s Alpha (α) for organizational trust subsequent to the completion of factor analysis. Factor 1 for Organizational Trust has six components, exhibiting a Cronbach’s Alpha of 0.913, and the researchers have designated this factor as “Reliance with Leader.” The second component has five elements with a Cronbach’s Alpha of 0.816, designated as “Harmony,” while the third factor, which achieved a Cronbach’s Alpha of 0.791 is termed as “Leader’s Behavior.” All criteria were redefined according to prior research pertinent to the current investigation. Therefore, factors 1, 2, and 3 were utilized for subsequent analysis correspondingly.

**TABLE 2:** Reliability values of organizational trust after computing factor analysis.

Organizational trust	Total of items	Cronbach’s alpha (α) after factor analysis
Factor 1 reliance with leader	6	0.913
Factor 2 harmony	5	0.816
Factor 3 leader’s behavior	4	0.791

#### 4.2.2 Factor analysis for spiritual leadership

Table 3 below illustrates the Cronbach’s Alpha result for Spiritual Leadership after computing factor analysis. the reliability test results spotted that only Factor 1 and Factor 2 consist with 6 and 4 items, respectively and achieved a reliable Cronbach’s Alpha value. The researchers retained the designation of altruistic love and hope for these elements. The other components exhibited a Cronbach Alpha value below 0.7, indicating insufficient reliability for subsequent investigation.

**TABLE 3:** Reliability values for spiritual leadership after factor analysis.

Spiritual leadership	No. of items	Cronbach’s alpha (α) after factor analysis
Factor 1 altruistic love	6	0.853
Factor 2 hope	4	0.866
Factor 3 (not reliable)	3	0.635
Factor 4 (not reliable)	2	0.521

Based on the result of Factor Analysis, the research framework is redeveloped to include two dimensions of the spiritual leadership and three dimensions of organizational trust.

### 4.3 Multiple regression analysis

#### 4.3.1 The effect of altruistic love and hope on reliance leader

As shown in Table 4, the *R*^2^-value is 0.420, or 42%, which is noteworthy or significant as it exceeds 25% (Hair et al., 2010). This signifies the sufficiency of the R square value in assessing the correlation between the observed and predicted criterion variable (Hair et al., 2010). The regression analysis indicated that altruistic love and hope significantly predict reliance on a leader at a level of *P* < 0.05. The substantial value (p ≤ 0.05) and the Beta coefficients (β) indicate that both dimensions of Spiritual Leadership, namely altruistic love and hope, exert a significant and positive influence on reliance on the leader. Notably, hope demonstrates a greater impact than altruistic Love, as the β value for hope is 0.647, signifying that hope accounts for 64.7% of the variance in reliance on the leader.

**TABLE 4:** Multiple regression findings for independent variables (altruistic love and hope) and dependent variable (reliance with leader).

Dimensions	IV-DV
Altruistic love	0.109*
Hope	0.647*
*F*-value	Significant at F = 122.767
*R*^2^-value	0.42

Significant at *P < 0.05.

#### 4.3.2 The effect of altruistic love and hope on harmony

As shown in Table 5 the *R*^2^-value is 0.303, or 30.3%, which is notable as it exceeds 25% (Hair et al., 2010). This signifies the sufficiency of the R square value in assessing the correlation between the observed and predicted criterion variable (Hair et al., 2010). The regression analysis indicated that altruistic love and hope significantly predict harmony at a level of *P* < 0.05. Upon examining the substantial value (*p* ≤ 0.05) and Beta coefficients (β), it is evident that only hope exerts a significant and positive influence on harmony, with a β value of 0.551, indicating that hope accounts for 55.1% of harmony.

**TABLE 5:** Multiple regression results for independent variables (altruistic love and hope) and dependent variable (harmony).

Dimensions	IV-DV
Altruistic love	0.187
Hope	0.551*
*F*-value	Significant at F = 75.711
*R*^2^-value	0.303

Significant at *P < 0.05.

#### 4.3.3 The effect of altruistic love and hope on leader’s behavior

As shown in Table 6 the *R*^2^-value is 0.456, or 45.6%, which is noteworthy as it exceeds 25% (Hair et al., 2010). This signifies the sufficiency of the R square value in assessing the correlation between the observed and predicted criterion variable (Hair et al., 2010). The regression analysis indicated that altruistic love and hope significantly predict harmony at a level of *P* < 0.05. Upon examining the substantial value (*p* ≤ 0.05) and Beta coefficients (β), it is evident that only hope exerts a significant and positive influence on leaders’ behavior, with a β value of 0.675, indicating that hope accounts for 67.5% of leaders’ behavior.

**TABLE 6:** Multiple regression results for independent variables (altruistic love and hope) and dependent variable (leader’s behavior).

Dimensions	IV-DV
Altruistic love	0.002
Hope	0.675*
*F*-value	Significant at F = 146.210
*R*^2^-value	0.456

Significant at *P < 0.05.

## 5 Discussion and conclusion

### 5.1 The effect of spiritual leadership on organizational trust in public university

As maintained by Fourie and Poggenpoel (2016), lack of trust has currently emerged as a primary problem in the public sector. Consequently, the senior management of every public sector body, including public university, exerts considerable effort to prevent or, at the very least, mitigate employee wrongdoing, particularly among officers. Consequently, their confidence in the organization will diminish. This is attributable to executives who engage in misbehavior being unreliable and incapable of fulfilling corporate objectives. Furthermore, the character of leaders, encompassing honesty and fairness, is often concealed. Leaders at public universities concern for personnel benefits and rights; nonetheless, the wellbeing of the staff is not adequately addressed. The leader’s activities are mostly focused on their popularization. The absence of trust, integrity and equity demonstrated by public university’s management engenders distrust among the personnel toward the organization.

This study applies multiple regression analysis to investigate the effect of spiritual leadership on organizational trust. The results demonstrate that both dimensions of spiritual leadership significantly influence employees’ trust in their leaders. Specifically, it shows that public university support staff believe leaders must consistently prioritize their subordinates’ wellbeing, rights, and benefits, while also maintaining transparent communication as promised. These results were aligned with the work of Terzi et al. (2020). At the public university, officers must be committed to the welfare of their subordinates to earn their trust. It is widely recognized that working at a public institution can be demanding and sometimes chaotic, as all tasks must be completed accurately and according to established rules, regulations, and policies, often within tight deadlines. Furthermore, staff must meet the diverse needs and demands of various stakeholders. Officers must manage these challenges without diminishing their subordinates’ morale or motivation through undue pressure. Instead, they should consistently encourage their team by recognizing their efforts, ensuring resources are sufficient, accessible, and providing clear and accurate information. The study highlights that open communication is crucial for subordinates to view their officers as trustworthy and reliable. Therefore, concern about employees’ welfare and rights delivering accurate information and fostering open communication this leader can build confidence in their team’s ability to achieve clear objectives, while simultaneously strengthening trust between leaders and subordinates.

The study also found that only hope had a significant impact on harmony in the other regression results as shown in Table 7. The likely rationale for this outcome is that public university support staff trust their leader, who is effectively guiding the university toward its goals through transparent communication. This approach elucidates the public university’s objectives and direction, fostering positive sentiments, interests, opinions, purpose, and values essential for maintaining a harmonious work environment. This aligns with the findings of Faruk et al. (2019b), which indicate that leaders who exhibit spirituality in their leadership can cultivate trust among their subordinates. In public universities, both official meetings and informal discussions conducted routinely must be grounded in factual information. Items not included in the meeting agenda should be disregarded. Leaders must also eschew the demeanor of “talking without taking action.” Monitoring is fundamental to assessing the efficacy and accomplishment of assigned activities. These may alleviate doubt and ambiguity among subordinates regarding the university’s capacity to fulfill its goals, aims, and vision. Minimizing uncertainty and ambiguity will enhance the university’s working environment and foster a more harmonious connection between subordinates and superiors.

**TABLE 7:** Summary of hypothesis test.

	Hypothesis	Results
H1a	There is a significant effect between altruistic love and reliance with leader.	Supported
H1b	There is a significant effect between hope and reliance with the leader.	Supported
H2a	There is a significant effect between altruistic love on harmony.	Not supported
H2b	There is a significant effect between hope on harmony.	Supported
H3a	There is a significant effect between altruistic love on a leader’s behavior.	Not supported
H3b	There is a significant effect between hope on leader’s behavior.	Supported

This study also shows that, altruistic love not enough to emphasis the team harmony, unlike the hope plays a crucial role in shaping a leader’s actions and positively embark the harmony among the team. As noted earlier, a leader’s behavior reflects key values such as integrity, honesty and fairness, while hope is defined by subordinates’ expectations regarding the university’s ability to achieve its goals and vision. When subordinates lack confidence in the organization’s success, their motivation to carry out their responsibilities diminishes. They will see that their employment and contributions to the company will culminate in a dead end. Consequently, individuals may perceive their efforts as futile, despite their desire to provide ideas to management. To rectify this problem, leaders must operate with integrity, ensuring transparency is paramount and that all choices adhere to relevant rules, regulations, and policies. Leaders must also exhibit integrity in their decision-making processes. They will refrain from making any decisions that may result in personal gain. Additionally, leaders must ensure fairness and equity in their treatment of subordinates, avoiding any form of discrimination in their interactions.

### 5.2 Conclusion

Organizational trust is regarded as a crucial priority inside a business, as this climate fosters leaders’ competence, transparency, integrity, employee welfare, and dependability. Organizations are confronted with a growing desire to address employee-employer relationships in order to enhance mutual obligation. This research aims to test the impact of spiritual leadership on organizational trust among administrative and support personnel at a public university, based on prior literature and findings. The empirical findings validated 4 out of 6 hypotheses, addressing all core research questions despite noted limitations, and corroborated the principal conceptual framework behind the current study. Nonetheless, the research results aligned with other prior qualitative investigations conducted. The study developed and validated the model that predict and interpret the trust in organization by the spiritual leadership. These findings shed the light on the value of adopting spiritual leadership strategies among organizations in academic public sector in Malaysia. The senior management HR departments can utilize this model to enhance the overall academic staff performance, and to tackle the current barriers that inhibit the universities progress. However, the cross sectional design consider on of limitation of this study, the further mix-methodology and multi-phase investigations and research should be conducted to utilize this model in different sectors, specially in the academic sector in Malaysian universities to highlight the impact of spiritual leadership on trust factor. Additionally, its highly recommended to conduct case studies in specific academic sector to support this finding.

## Data Availability

The raw data supporting the conclusions of this article will be made available by the authors, without undue reservation.

## References

[B1] Abbasi A. Wan Ismail W. K. (2023). Linking organizational citizenship behavior and organizational trust towards reducing workplace deviance behavior in higher education. *Cogent Soc. Sci.* 9:2157538. 10.1080/23311886.2022.2157538

[B2] Ayduğ D. (2025). *The level of academic identities of faculty members predicting their organizational trust.* Manila: The Asia-Pacific Education Researcher., 10.1007/s40299-025-00970-6

[B3] Basit A. Duygulu E. (2018). Extending the chain of relationships between organizational trust and intention to remain among self-initiated academic expatriates. *Int. J. Acad. Res. Bus. Soc. Sci.* 8 1372–1385. 10.6007/IJARBSS/v8-i9/4702

[B4] Blum J. Manning N. Srivastava V. (2012). *Public sector management reform: Toward a problem-solving approach (English). Economic premise*, Vol. 100. Washington, DC: World Bank Group.

[B5] Chen C.-J. Huang J.-W. (2016). Effects of organizational trust on organizational learning and creativity. *Eur. J.Maths. Sci. Technol. Educ.* 12 1675–1686. 10.12973/eurasia.2016.1551a

[B6] Chen L. Wen T. Wang J. Gao H. (2024). the impact of spiritual leadership on employee’s work engagement: A study based on the mediating effect of goal self-concordance and self-efficacy. *Int. J. Ment. Health Promot.* 24 69–84. 10.32604/ijmhp.2022.018932

[B7] Cheng C.-Y. Wang Y.-C. (2025). Organizational trust and employee work outcomes: A moderated mediation model. *Curr. Psychol.* 44 6565–6578. 10.1007/s12144-025-07626-0

[B8] Constantin I. I. (2024). The impact of spiritual leadership on the performance of employees’ creative services. *Ann. Econ. Ser.* 3 253–259.

[B9] Conway J. M. (1999). Distinguishing contextual performance from task performance for managerial jobs. *J. Appl. Psychol.* 84 3–13. 10.1037/0021-9010.84.1.3

[B10] Daud Z. Elsaman H. A. Ahmad R. Mutazam M. Sallehuddin M. R. Haron N. (2024a). Influence of ethical management on politics in performance appraisal: Development and validation of a causal model. *Int. J. Adv. Appl. Sci.* 11 48–55.

[B11] Daud Z. Elsaman H. A. Mutazam M. Sallehuddin M. R. Haron N. Zainol Abidin M. A. (2024b). Does the leadership style influence the office politics in performance appraisal? Empirical study from Malaysian financial sector. *Edelweiss Appl. Sci. Technol.* 8 1071–1082. 10.55214/25768484.v8i5.1807

[B12] Daud Z. Elsaman H. A. Sallehuddin M. R. (2023). The office politics error as a new dimension in performance appraisal implementations: A case study and conceptual model in Malaysian financial sector. *Acta Innov.* 48 48–60. 10.32933/ActaInnovations.48.4

[B13] Daud Z. Ismail S. A. Rashdan M. S. Husin M. F. (2018). Office politics as an element of office ecosystem. *J. Soc. Sci. Res.* 2018 547–552. 10.32861/jssr.spi6.547.552

[B14] Daud Z. Saiful Azizi I. Mohd Rashdan S. Ahmad R. (2019). Office ecosystem: The effect of personal attribute on employees’ perception on office politics. *Int. J. Innov. Creativ. Change* 5 502–513.

[B15] Daud Z. Saiful A. Sallehuddin M. R. (2021). The effect of personality on office politics perception: An experience from malaysian government agencies. *Int. J. Innov. Creativ. Change* 15 469–482.

[B16] Deci E. L. Ryan R. M. (2000). Self-determination theory and the facilitation of intrinsic motivation, social development and well-being. *Am. Psychol.* 55 68–78. 10.1037//0003-066x.55.1.68 11392867

[B17] Elsaman H. A. Daud Z. Salehuddin M. R. (2024a). Fly me to the moon of business performance; elevating the organisational performances trough organisational commitment and the mediating role of change management. *Edelweiss Appl. Sci. Technol.* 8 2168–2180. 10.55214/25768484.v8i4.1590

[B18] Elsaman H. A. Dayanandan R. Daud Z. Saleh A. A. (2024b). Navigating Fintech innovation: Performance, trust and risk factors in UAE’s banking sector. *J. Eastern Eur. Cent. Asian Res.* 11 332–341. 10.15549/jeecar.v11i2.1569

[B19] Elsaman H. A. El-Bayaa N. Kousihan S. (2022). Measuring and validating the factors influenced the SME business growth in Germany—descriptive analysis and construct validation. *Data* 7:158. 10.3390/data7110158

[B20] Esfahani S. T. Sedaghat S. (2015). The relationship between spiritual leadership and vertical organizational trust. *J. Sci. Res. Dev.* 2 166–171.

[B21] Faruk S. Daud Z. Ismail S. A. (2019a). Organizational justice and psychological employment contract breach: The mediating effect of trust. *Opcion* 35 113–128.

[B22] Faruk S. Daud Z. Ismail S. A. (2019b). Perceived organizational support and psychological employment contract breach: A proposed model on the mediating effect of trust. *Int. J. Recent Technol. Eng.* 8 314–318. 10.35940/ijrte.B1054.0782S319

[B23] Fourie D. Poggenpoel W. (2016). Public sector inefficiencies: Are we addressing the root causes? *South Afr. J. Account. Res.* 31 1–12. 10.1080/10291954.2016.1160197

[B24] Fry L. W. (2003). Toward a Theory of Spiritual Leadership. *Leadersh. Q.* 14 693–727. 10.1016/j.leaqua.2003.09.001

[B25] Fry L. W. Cohen M. P. (2009). Spiritual leadership as a paradigm for organizational transformation and recovery from extended work hours culture. *J. Bus. Ethics.* 84 265–278. 10.1007/s10551-008-9695-2

[B26] Fry L. W. Vitucci S. Cedillo M. (2005). Spiritual leadership and army transformation: Theory, measurement, and establishing a baseline. *Leadersh. Q.* 16 835–862. 10.1016/j.leaqua.2005.07.012

[B27] Garg N. Jalan S. (2024). Spiritual leadership research: Past, present, and future using bibliometric analysis. *J. Religion Health* 64 999–1030. 10.1007/s10943-024-02178-2 39557819

[B28] Ghasemy M. Hussin S. Daud S. (2022). Lecturers’ interpersonal trust in peers, job performance, and organizational citizenship behavior: Examining the mediating role of positive affect during the COVID-19 pandemic utilizing the PLS-SEM estimator. *Int. J. Product. Perform. Manag.* 73 1996–2015. 10.1108/IJPPM-10-2022-0523

[B29] Guillen L. Mayo M. Korotov K. (2015). Is leadership a part of me? A leader identity approach to understanding the motivation to lead. *Leadersh. Q.* 26 802–820. 10.1016/j.leaqua.2015.05.001

[B30] Hair J. F. Black W. C. Babin B. J. Anderson R. E. Tatham R. L. (2006). *Multivariate data analysis*, 6th Edn. Hoboken, NJ: Pearson Prentice Hall.

[B31] Hair J. F. Black W. C. Babin B. J. Anderson R. E. Tatham R. L. (2010). *Multivariate data analysis*, 7th Edn. Hoboken, NJ: Pearson Prentice Hall.

[B32] Hair J. F. Sarstedt M. Ringle C. M. Mena J. A. (2012). An assement of the use of partial least squares structural equation modeling in marketing research. *J. Acad. Market. Sci.* 40 414–433. 10.1007/s11747-011-0261-6

[B33] Hassan A. Ahmed F. (2011). Authentic leadership, trust and work engagement. *Int.l J. Hum. Sci.* 8 164–171.

[B34] Hu Y. Zhang L. (2023). The effect of spiritual leadership on proactive customer service performance: The role of psychological empowerment. *Hum. Soc. Sci. Commun.* 10:273. 10.1057/s41599-023-02273-x

[B35] Ismail S. A. Daud Z. Zaini A. F. A. (2019). The influence of leadership in the relationship of perceived perception of organizational politics in Islamic financial organization based at Malaysia. *Jurnal Pengurusan* 56 1–19.

[B36] Jahroni S. Dwijanto A. Kistyanto A. (2024). Spiritual leadership, religiosity, and change management effectiveness: A study in educational organizations. *J. Pendidikan Dan Pembelajaran* 5 1069–1082. 10.62775/edukasia.v5i1.942

[B37] Jaškevičiūtė V. Šilingienė V. (2021). The relationship between employee well-being and organizational trust in the context of sustainable human resource management. *SHS Web Confer.* 92:04011. 10.1051/shsconf/20219204011

[B38] Joo B.-K. Yoon S. K. Galbraith D. (2023). The effects of organizational trust and empowering leadership on group conflict: Psychological safety as a mediator. *Organ. Manag. J.* 20 4–16. 10.1108/OMJ-07-2021-1308

[B39] Kark R. Shamir B. (2023). The dual effect of transformational leadership: Priming relational and collective selves and further effects on followers. *Transform. Charismat. Leadersh.* 2 77–101. 10.1016/B978-0-12-398456-2.00002-2

[B40] Kriejcie R. Morgan D. (1970). Determining sample size for research activities. *Educ. and Psychol. Meas.* 30 607–610. 10.1177/001316447003000308

[B41] Lee J. Kim S. (2022). The mediating role of employee organizational trust in shaping job satisfaction and turnover intention. *J. Manag. Educ.* 46 563–584. 10.1177/23294884221081838

[B42] Li X. Wang Y. (2023). Exploring the influence of spiritual leadership on proactive service performance in the hospitality industry. *Acta Psychol.* 250:103034. 10.1016/j.actpsy.2025.104721 39823990

[B43] Locke E. A. Shaw K. N. Saari L. M. Latham G. P. (1981). Goal setting and task performance: 1969-1980. *Psychol. Bull.* 90 125–152. 10.1037/0033-2909.90.1.125

[B44] Mayer R. C. Davis J. H. Schoorman F. D. (1995). An integrative model of organizational trust. *Acad. Manag. Rev* 20 709–734. 10.2307/258792

[B45] Moghavvemi S. Sharabati M. Klobas J. E. Sulaiman A. (2018). Effect of trust and perceived reciprocal benefit on students’ knowledge sharing via Facebook and academic performance. *Electron. J. Knowledge Manag.* 16 23–35.

[B46] Mutahar Y. Farea M. M. Abdulrab M. Al-Mamary Y. H. Alfalah A. A. Grada M. (2022). The contribution of trust to academic knowledge sharing among academics in the Malaysian research institutions. *Cogent Bus. Manag.* 9:2038762. 10.1080/23311975.2022.2038762

[B47] Mutazam M. Daud Z. (2021). The effect of monitoring on authentic leadership amongst administrators in malaysian public universities. *Glob. J. A Thaqafah* 11 49–61.

[B48] Rahmatika A. N. Ma’arif S. Kholifah S. (2022). The effect of spiritual leadership and psychological empowerment on employee performance. *Nidhomul Haq J. Manaj. Pendidikan Islam* 7 123–135. 10.31538/ndh.v7i3.2678

[B49] Samul J. (2024). *Linking spiritual leadership with other leadership concepts: A literature review of four decades.* Thousand Oaks, CA: SAGE Open, 10.1177/21582440241252402

[B50] Saraih U. N. Mohd Karim K. Abu Samah I. H. Amlus M. H. Abashah A. N. (2018). Relationships between trust, organizational justice and performance appraisal satisfaction: Evidence from public higher educational institution in Malaysia. *Int. J. Eng. Technol.* 7 602–606. 10.14419/ijet.v7i2.29.13983

[B51] Sekaran U. (2003). *Research methods for business: A skill building approach.* New York, NY: John Wiley and Sons.

[B52] Sekaran U. Bougie R. (2010). *Research methods for business: A skill building approach*, 5th Edn. Hoboken, NJ: John Wiley and Sons.

[B53] Shockley-Zalabak P. Ellis K. Winograd G. (2000). Organizational trust: What it means, why it matters. *Organ. Dev. J.* 18 35–48.

[B54] Sihombing A. Sari E. Abbas H. (2024). Servant leadership’s impact on trust, commitment, and performance in higher education. *Int. J. Eval. Res. Educ.* 13:1703. 10.11591/ijere.v13i3.27297

[B55] Subhaktiyasa P. G. Agung A. A. G. Jampel I. N. Dantes K. R. (2023). Spiritual leadership in educational organization: A systematic literature review. *J. Law Sustain. Dev.* 11:e722. 10.55908/sdgs.v11i5.722

[B56] Terzi R. Gocen A. Kaya A. (2020). Spiritual leaders for building trust in the school context. *Eur. J. Educ. Res.* 86 135–156.

[B57] Tsai Y. Wu S. W. (2024). Organizational culture and trust affect the team-based practice and job satisfaction of nurse practitioners in acute care practices. *Nurs. Open* 11:2049627. 10.1155/2024/2049627 40224855 PMC11918914

[B58] Tzafrir S. S. Dolan S. L. (2004). Trust me: A scale for measuring manager-employee trust. *Manag. Res.* 2 115–132. 10.1108/15365430480000505

[B59] Vedula S. B. Agrawal R. K. (2023). Mapping spiritual leadership: A bibliometric analysis and synthesis of past milestones and future research agenda. *J. Bus. Ethics* 189 301–328. 10.1007/s10551-023-05345-0

[B60] Vesudevan M. Abdullah Z. (2024). Integrating sustainable leadership in Malaysian higher education: Effective strategies for implementation and impact. *Malaysian Rev.* 9 45–58. 10.31893/multirev.2025115

[B61] Woo S. Y. Kim S. (2020). Determination of cutoff values for biomarkers in clinical studies. *Precis. Future Med.* 4 2–8. 10.23838/pfm.2019.00135

[B62] Yıldız M. L. Şimşek Y. (2019). Organizational trust, employee commitment, and job satisfaction in Turkish physicians. *Nurs. Ethics* 26 831–847. 10.1177/0969733017722825 28893160

